# Patient and professional perspectives on physical activity promotion in routine cancer care: a qualitative study

**DOI:** 10.1186/s12913-024-11480-4

**Published:** 2024-09-30

**Authors:** Kadia Saint-Onge, Jany St-Cyr, Isabelle Doré, Lise Gauvin

**Affiliations:** 1 Department of Kinesiology, Faculty of Medicine, Université Laval, Quebec, QC Canada; 2 Department of Social and Preventive Medicine, School of Public Health, Université de Montréal, Montréal, QC Canada; 3 Centre de recherche du Centre hospitalier de l’Université de Montréal, Montréal, QC Canada; 4 Department of Psychology, Université du Québec à Montréal, Montréal, QC Canada; 5 School of Kinesiology and Physical Activity Sciences, Faculty of Medicine, Université de Montréal, Montréal, QC Canada

**Keywords:** Physical activity, Cancer survivorship, Health promotion, Health systems, Community

## Abstract

**Backgrounds:**

Physical activity is associated with many benefits in reducing cancer symptoms and treatments side effects. Yet, studies consistently show that knowledge about physical activity is under-promoted among people diagnosed with cancer. Therefore, we aimed to contribute to filling this gap by ascertaining patient and professional perspectives regarding physical activity promotion.

**Methods:**

This study took place in Montreal, Canada. We conducted individual, semi-structured interviews with cancer patients who participated in a physical activity program and professionals working in the healthcare system. Participants had to be aged over 18 years, be able to communicate verbally in either English or French, and consent to an audio-recorded interview. A hybrid deductive-inductive approach to content analysis was applied to analyze interview transcripts using Dedoose and Microsoft Excel software.

**Results:**

Our sample comprised 21 patients (76.2% women) and 20 professionals (80% women). We identified 24 factors (barriers, facilitators, and improvement suggestions) influencing physical activity promotion across organizational, community, and social levels. Results suggest that to improve physical activity promotion in cancer care, it is necessary to showcase exercise specialists as a healthcare resource, to champion for this change within health organizations, to develop partnerships between public and private sectors of the health and fitness industries, and to reassess social norms concerning cancer survivorship and treatment.

**Conclusion:**

These findings shed light on the gaps and the bright lights in physical activity promotion for people diagnosed with cancer across numerous levels.

## Introduction

In Canada, two in five people will be diagnosed with cancer in their lifetime [1]. Cancer is the primary cause of premature death among Canadians [1], and its treatment often causes short- and long-term side effects. Much attention has been directed towards physical activity’s role in reducing cancer mortality and incidence [2–4]. More recent work has focused on physical activity benefits in reducing cancer symptoms and treatments side effects. That is, physical activity decreases cancer-related fatigue, depressive and anxious symptoms, improves quality of life and perceived physical function, and is associated with increased survival rates for some cancer types [5–9]. To achieve these health benefits, current evidence-based recommendations for people diagnosed with cancer suggest aiming for at least 90 min of aerobic activity at moderate intensity as well as two strength-training sessions per week [7]. Recommendations also emphasize the importance of avoiding inactivity [5]. Nevertheless, data show that people diagnosed with cancer do not reach these recommendations [10, 11]. Despite physical activity benefits, studies consistently show that it is under-promoted in clinical oncology settings, prompting scholars to declare that “Current practice is failing those diagnosed with cancer” (p. 21) [8]. This study aims to contribute to filling this gap by ascertaining patient, organizational and professional perspectives on how to promote physical activity among cancer patients.

In accordance with the World Health Organization’s definition of health promotion, physical activity promotion can be described as the process of enabling people to increase control over, and to improve, their physical activity [12]. The WHO further specifies that promotion “*not only embraces actions directed at strengthening the skills and capabilities of individuals*,* but also action directed towards changing social*,* environmental*,* and economic determinants of health so as to optimize their positive impact on public and personal health.*” (Nutbeam & Muscat, 2021, p. 1580). Physical activity promotion thus includes actions such as building infrastructure for guided exercise within clinical settings. It also includes one-on-one intervention such as the “5A”s”: Ask, Advise, Assist and Arrange” put forward by the Agency for Healthcare Research and Quality [13].

In keeping with that definition, guidelines for the implementation of chronic disease management [14] underscore the importance of assessing an encompassing list of factors when implementing physical activity promotion among people diagnosed with cancer. These factors range across numerous levels including individual (e.g., patient readiness), health professionals (e.g., practices and evidence), organizational (e.g., resources in the clinical setting), community (e.g., availability of programs and services), and the greater social environment (e.g., social norms) [5, 15–23]. Evidence suggests that an effective promotion strategy should address key ingredients at each of these levels. Key ingredients at the individual level include patients’ readiness, interest, and socioeconomic resources to engage in physical activity as well as their knowledge base about the subject [5, 16, 18–20]. For health professionals, the promotion of physical activity is facilitated by clinicians’ knowledge of and ease in promoting physical activity to people diagnosed with cancer, their access to promotional material, their knowledge of referral resources, and by solid program evaluation data [5, 16–18, 20, 22, 24]. At the organizational level, providing access to continuous healthcare training about physical activity and cancer, and support from organizational leadership and colleagues in hospital settings facilitate the implementation of physical activity promotion [5, 15, 17–22]. At the community level, an effective promotion strategy includes financial, geographic, and sociocultural access to physical activity outlets (private gyms, community clubs, etc.) or clinical physical activity programs [5, 18–20]. Finally, examples of social environment ingredients include government policies to support physical activity promotion including funding physical activity programs for people diagnosed with cancer, or privately funded initiatives to offer such programs [17, 20].

There is consensus that to maximize physical activity benefits for people diagnosed with cancer, physical activity promotion must be integrated into multiple levels of care targeting the multi-level factors such as those mentioned above holistically [25–27]. Yet, most evidence about the factors affecting implementation of physical activity promotion in clinical oncology settings comes from patients expressing the role of individual level factors and to a lesser extent health professionals expressing the role of organizational level factors [19]. That is, current knowledge underrepresents organizational stakeholders’ perspectives [21], organizational factors influencing program delivery [18], as well as factors occurring at the community or at the societal levels. To contribute to filling this gap and replicating existing findings, we ascertained patient and professional perspectives regarding barriers, facilitators, and suggestions to promote physical activity.

## Methods

### Design

A qualitative research design was used to identify barriers and facilitators to the implementation of physical activity promotion as well as suggestions for its improvement [28]. A deductive-inductive content analysis approach was chosen because it is well suited for preparing, organizing, and reporting a large corpus of complex data [28, 29]. One-on-one semi-structured interviews were conducted with patients and with health professionals.

### Recruitment and ethical considerations

Participants had to be aged over 18 years, be able to communicate verbally in either English or French, and consent to an audio-recorded interview. They could not participate if they did not meet these criteria, or the ones described below. Two sampling strategies were used to recruit people diagnosed with cancer and health professionals respectively. First, to facilitate recruitment of people diagnosed with cancer, we randomly selected sixty patients (30 men, 30 women) who had or were participating in a physical activity program geared specifically for people diagnosed with cancer in the previous 2 years. This program was offered at no cost by the Virage Foundation, which operates within a large hospital center (CHUM, Centre hospitalier de l’université de Montréal) in the city of Montreal, Canada. Using the sixty randomly selected subsample of program attendees, we contacted potential participants sequentially via telephone and offered them to participate in the study. As for the professionals, the research team identified and invited via email known professionals as well as organizational managers and leaders having prior experience in oncology healthcare or providing physical activity services within a hospital environment. We solicited professionals with experience in promoting or providing physical activity to people diagnosed with cancer as well as those with experience in developing or providing cancer care.

This study is part of a larger project detailed elsewhere [30]. Potential participants were provided the study information and consent form, time to review it, and the opportunity to ask questions. Study participants provided written informed consent to audio recorded interviews. The CHUM Research Center Ethics Board reviewed and approved the study protocol (# 17.238).

### Procedures for data collection

Our research team was composed entirely of women. It included five research assistants (four graduate students in public health (M. Sc.), community psychology (Ph. D., KSO), social psychology (B. Sc., JSC), and physical activity sciences (M. Sc.) as well as a research professional (MD). Lastly it also included two senior level researchers with extensive expertise in physical activity science as well as social and preventive medicine (Ph. D. University Professors, ID and LG). The research team adopted an epistemological position in which we aimed for objectivity recognizing that our work is influenced by the values conveyed in health promotion. These include benevolence, equity, usefulness and efficacy, transparency, common good, as well as empowerment.

Semi-structured individual interviews were conducted face-to-face at the hospital center before the COVID pandemic onset and via teleconference or telephone while confinement policies were in effect. The senior authors developed the interview guides (see Doré et al., 2022) for complete interview guides). The interview guides were geared to obtain information about the physical activity program as a part of the larger research project, but specific questions also targeted participants’ perceptions on existing promotion of physical activity to people diagnosed with cancer inside and outside the hospital setting as well as its improvement. Three research assistants trained in interviewing techniques conducted interviews with 21 patients and 20 professionals between June 3, 2019, and October 26, 2020. Data collection ended once theoretical saturation was achieved [31]. That is, we ceased to interview participants once no new information emerged and the data collected satisfactorily met the study objectives. On average, interviews lasted 35.05 min (MD = 35.00, SD = 10.48, Min = 17.00, Max = 57.00). They were digitally recorded then transcribed verbatim by an external service provider. Research assistants were tasked with keeping a journal of field notes which were discussed during weekly team meetings. To ensure participants’ anonymity, minimal sociodemographic data were collected, and data were stored on a secure server.

### Data analysis

Interview transcripts were analyzed using a hybrid deductive-inductive approach to content analysis [28, 29, 32] with Dedoose and Microsoft Excel software. In the first *preparation* phase of coding [29], all researchers participated in a three-part training on content and thematic analysis to harmonize approaches, knowledge, and previous experiences with qualitative analysis. Four research assistants then coded deductively using an empirically based tool for health promotion implementation checklist as a guide. The “Tailored Implementation for Chronic Diseases” (TICD) checklist [14] served as the initial deductive coding grid [28]. The TICD checklist [14] covers a range of ecological factors influencing the implementation of promotion initiatives for chronic disease such as cancer. Specifically, it includes seven main domains relevant to implementing physical activity promotion in cancer healthcare practice: patient factors, individual health professional factors, professional interactions, capacity for organizational change, guideline factors, incentives and resources, as well as social, political, and legal factors. These domains served as the initial codes whereby research assistants applied them to relevant interview excerpts whilst also looking for new emerging codes not represented in the TICD checklist but answering our research question. Using the TCDI checklist as a coding grid facilitated the identification and labeling of multi-level facilitators and barriers.

In the second *organization* phase of coding [29], the two first authors abstracted initial codes into broader themes that better represented the data (organizational, community, and sociocultural factors influencing promotion implementation). The first author then refined codes and grouped them as a function of perceived factors that hinder (barriers), help (facilitators), or could improve (suggestions) physical activity promotion for people diagnosed with cancer. For R*eporting*, the third and last phase of coding, the research team met regularly to discuss and refine themes and sub-themes as well as to select illustrative quotes to depict each of them.

To support coding rigor and reproducibility, research assistants met weekly to discuss their coding strategies and challenges. For member-checking purposes [28], we presented preliminary data to professionals having participated in the study interviews and integrated their feedback.

## Results

Our sample includes twenty professionals (80% women) working in oncology, nursing, cardiology, kinesiology, public health, and management as well as twenty-one patients (76.2% women) in cancer care of various sites: breast, prostate, thyroid, lung, and others. Below, we describe perceived barriers and facilitators as well as suggestions for improvement of physical activity promotion among people diagnosed with cancer as a function of three levels of analysis: (1) Organizational factors; (2) Community factors; (3) Sociocultural factors. Table 1 provides a detailed overview of these findings.

**Table 1:** List of barriers, facilitators, and improvement suggestions for physical activity promotion among people diagnosed with cancer as a function of organizational, community, and sociocultural factors

Factor Level	A. Barriers	B. Facilitators	C. Improvement Suggestions
**1. Organizational**	**1.A.1 Limited knowledge transfer to health professionals** • Lack of knowledge about physical activity and cancer • Limited comfort promoting physical activity to patients • Low knowledge of PA outlets in and outside of the hospital	**1.B.1 Qualified exercise professionals specializing in oncology** • Personalized and adapted plans by experts in the matter • Continuous monitoring • Facilitated transition from the hospital to the community • Creativity and adaptability	**1.C.1 Emphasise exercise professionals’ vital role in cancer care** • Integrate exercise specialists in treatment teams • More seamless interdisciplinary communication
	**1.A.2 Resource scarcity** • Physical space for exercise • Human Resources • Time available during consultation	**1.B.2 Championing from all concerned parties** • Clinicians modelling promotion to their peers • Clinical teams calling for clinical gymnasiums in oncology • Importance of lived experience in planning and promotion	**1.C.2 Develop tools and training opportunities** • Promotional material • Patient risk factor assessment checklist • Systematically expose new clinicians to PA promotion • Conferences for professionals • Informational “healthy hours” between patients and professionals
	**1.A.3 Constrained service offer** • Limited schedule • Differing modality preferences (group, individual, virtual) • Diversity and inclusion (age, cultural background, etc.)	**1.B.3 Interprofessional collaboration** • Access to patient data • Shared responsibility for physical activity promotion • Knowledge transfer to community resources	**1.C.3 Measure physical activity’s impact** • Short, medium, and long term • Research specific dosage and cancer sites
2. **Community**	**2.A.1 Sociodemographic inequities** • Limited access to specialized exercise specialists • Cost of PA outlets in general • Cost of parking near hospital facilities • Mostly reaching motivated, educated middle-class patients	**2.B.1 Interest from the private sector** • Potential for partnerships with private gyms • Medical clearance for insurance reimbursements	**2.C.1 Develop and nurture intersectoral collaboration** • Integrate public and private sectors • Explore innovative business models
	**2.A.2 Concerns about service availability and quality** • Labor shortage and high turnover • Difficult to monitor progress short and long term • Unknown professional standards and equipment sanitization	**2.B.2 Involvement of philanthropic organizations** • Decisional autonomy • Low or no-cost activities • Provide complementary as opposed to competitive services • Financial support from individuals in the community	**2.C.2 Guide turnkey programs** • Develop, implement, and maintain ready-made programs • Implement tele mentoring for exercise specialists
3. **Sociocultural**	**3.A.1 Social norms about cancer survivorship and physical activity** • Expectations of sedentariness when surviving cancer • Negative beliefs and attitudes towards physical activity • Gender-normative stereotypes about patients and physical activity	**3.B.1 Social support for participation in physical activity during cancer treatment** • Friends and family encouraging physical activity • General support during treatment	**3.C.1 Raise public awareness** • Engage in social media • Utilize tailored, inclusive messaging **3.C.2 Standardize universal physical activity cancer care** • Rethink family practice to promote physical activity long term • Develop a cancer-physical activity excellence center • Scale-up programs online province-wide **3.C.3 Fund physical activity programs in cancer care** • Individual responsibility • Insurance company incentives • Governmental responsibility
	**3.A.2 Transition back to routine in a demanding world** • Pressure to perform the same as pre-diagnosis • High-paced lives with delicate work-life balance • Parenthood before self-care	**3.B.2 Medical culture embracing adjuvant treatments** • Positive shift in clinician beliefs about physical activity benefits • Shift in medical culture towards prescribing less medication and more healthy lifestyle habits	
	**3.A.3 Lack of funding for physical activity in cancer care** • Preventive actions overlooked healthcare • Insufficient reimbursements for holistic treatment options • Lack of ownership for PA funding in government departments	**3.B.3 Population increasingly seeking health information** • Greater access to information via the internet • Patients are now asking clinicians more questions • Greater interest in complementary treatment options	

### 1. Organizational factors

This section presents the perceived factors influencing physical activity promotion among people diagnosed with cancer within the hospital setting. This includes factors influencing physical activity promotion such as professional practices, service provision (e.g., diversity and variety of physical activity programs), organizational resources, and functioning. Table 2 presents illustrative quotes describing each of these factors.

**Table 2:** Organizational level factors: quotes from patients (PAT) and professionals (PRO) describing barriers, facilitators, and improvement suggestions

A. Barriers	B. Facilitators	C. Improvement Suggestions
**1.A.1 Limited knowledge transfer to health professionals** I think we’re kind of helpless. We don’t know the resources. We don’t know what the person likes. We don’t know… And we don’t know how to do that right now. We don’t know where they are. So, it’s a whole lot of unknown. So, maybe if there was a program set up, maybe there would be a ripple effect. —PRO5	**1.B.1 Qualified exercise professionals specializing in oncology** It’s a 12-week program managed by kinesiologists who do a complete evaluation of the patient’s physical condition, taking into account their medical condition as well. They have access to the patient’s medical records, so they can really personalize the exercises and it’s group training. So, the whole group engagement effect, the gang, all that. —PRO5	**1.C.1 Emphasize exercise professionals’ vital role in cancer care** Indeed, I think that yes, to integrate kinesiologists with us from the start, and they too would have more information and perhaps more confidence or tools to know what to refer, where to refer. —PRO12
If the doctors were more aware, maybe they are aware, mine didn’t recommend it nor the surgeon, nor the specialist. I would have liked it to be posted somewhere. I didn’t see it. Maybe it was, it just wasn’t known. —PAT4	I know other trainers, but [the exercise specialists at Virage are really valuable resources]. Right from the start, for the combination they have of knowing the hardship we’re going through, the knowledge of the human body and of our illness, at least, minimally compared to others, and of physical training. That combination is very, very valuable. —PAT14	Most important is to have the professional human resources to take care of it: so, kinesiologists. I mean, I see kinesiologists. There may be other types, but I find that they, looking at interdisciplinary teams, we have them in the hospitals. So, it’s these positions [kinesiologists in oncology] that need to be funded. —PRO18
**1.A.2 Resource scarcity** In these environments often, there will be physical space issues. They’ll tell us that there is a lack of space. Financial barriers because it’s hard to quantify and it’s hard to validate a program to say that this program is effective because we’re in prevention all the time. As long as we’re not in curative care, as long as we don’t have tangible data on the benefits… —PRO9	**1.B.2 Championing from all concerned parties** I would tell others in my Monday … with others: ‘There’s this program: check it out!’ I’ve told several people: ‘They’re just not known but go!’ It’s not because—again, it’s not a big gym—it’s just that first, it forces you to go. Second, they give you a personalized program to follow up with you and that you’re able to do. I’m convinced that it’s a good program. Again, it was good for me. I can’t see [it not being good]. If it hadn’t been good, I wouldn’t have done it at all… But I would scale it up. —PAT3	**1.C.2 Develop tools and training opportunities** And you can’t count on doctors, who have so much to say and do. Especially when you’ve been working for 10 years, they don’t do it. To change a behavior, you really have to give a tool to the patients, a sheet where it’s written. And follow up at some point, send another sheet at some point by mail. It could be like that with testimonials of patients who tell their story and who say that it saved them pain. It’s concrete: there are people who say that just doing exercise is good for your pain. —PAT7
I think there were like two or three times when there were people coming to play badminton at the same time. I think the room was like shared. So that was like [disappointed tone] ‘… okay…’ because we got that it was a shared room but… Let’s just say that it was less pleasant because it was noisier, and we didn’t have all the space we usually had. —PAT6	Our teams are certainly closer [to recommending physical activity] now because you see, in the last phase of [the hospital center], we were asked to have a gym for that clientele there. And we’re going to do it. And it’s because there’s pressure … but positive pressure from the clinical teams. —PRO18	You have to be trained in what you’re going to do so that it’s not too much or too little. And you have to be careful of the professionals who are going to treat. They have to know that they’ll be facing someone who has cancer or who has had cancer. It’s not trivial. Patients are going to relapse, patients will go through complications, patients are going to die. If you get someone to do physical activity and you find out three days later that they’ve died, uh… you’re going to have to deal with that. […] They have to have oncology training modules, a minimum of oncology culture and the issues. —PRO8
**1.A.3 Constrained service offer** Yeah, I looked into it a little bit actually, I would have liked that. For example, I know that the Breast Cancer Foundation offers yoga sessions, or things like that. Yeah, I would have liked to take part in that, but it was becoming complicated to get there and it was tiring. I couldn’t. I couldn’t go too many times a week, even though I had to go for treatments and blood tests, and that was already a lot. I had chemo every week in those days. It came quickly. —PAT5	**1.B.3 Interprofessional collaboration** I was saying earlier that the fact that a patient who wasn’t physically active before, all of a sudden, has a serious illness, well their first reflex, isn’t to say ‘I’m gonna go work out’! So, if there are other professionals who say ‘Listen, it’s very important that you be active, the people to go see who are going to help you do that are the kinesiologists [exercise specialists in Canada]’. I think it’s crucial for the patient’s confidence in physical activity. —PRO2	**1.C.3 Measure physical activity’s impact** The impact measurement it’s one of the weaknesses of teams. […] they want to implement new programs, innovations like Virage, and they don’t know how to. They implement them, they don’t think about how to measure the impact from the start. Are we really improving care? Are we really improving the return to work, the return to their living environment. Are we really improving certain conditions? So, they don’t think about it from the conception. They don’t know how to necessarily. Hence the importance of having an interdisciplinary team to be able to respond to this obstacle. So, the how is done: how to set up, how to measure. In general, the people who start programs are clinicians and researchers. The managers aren’t always very involved, nor are the decision makers, and I’m talking about the Ministry. So, that means that we build pilot projects without having thought about how to scale up these programs. —PRO15
And I don’t know if there are also groups of young people and groups of older people. I think it’s important. When a young person arrives with a lot of older people, they don’t identify to […] I had been to another center that gave activities for people with cancer. But it wasn’t very young there. I arrived there with people of 65, 70 [years] maybe more and they were more into yoga or things like that. I said, “Oh no!” It didn’t click with me. It was depressing enough to have cancer that to see myself, with older people or in the process, to see that I’m going to get old too… It was rough. —PAT7	I think a lot of people don’t know what a *kinesiologist* is, what they do. But they must not know that there is a program for physical conditioning. […] At the same time, we talked about it and we said we would put signs everywhere, but that often doesn’t change much. It’s really done by us—by the nurse in the treatment rooms, by the doctors through word of mouth. —PRO11	Well, what we have within our reach right now, I think, is a solid final evaluation with the patients, a way to take stock of what we’ve done together and plan for the next step. I think that’s vital. Not just letting people go at the end but to do a minimum of follow-up with them. —PRO2

#### 1.A. Barriers

Both professionals and patients stated that clinicians need to be better prepared to promote physical activity to people diagnosed with cancer. **1.A.1 Limited knowledge transfer to health professionals** included exposure to scientific literature on related benefits as well as lack of knowledge concerning how and where to refer patients. Professionals and a few patients emphasized that **1.A.2 Resource scarcity** greatly reduced physical activity promotion for people diagnosed with cancer. Lack of physical space and human resources to host the program were especially commonly mentioned by exercise specialists and managers. Clinicians often mentioned lacking time to address and promote physical activity. Lastly, professionals and patients noted characteristics that **1.A.3 Constrained service offer**. These characteristics were mainly but not exclusively mentioned by patients. Participants mentioned elements such as physical activity program time slots conflicting with treatment or daily life, and the need for more peer support and diversity in group makeup. For example, some participants mentioned the lack of groups exclusively for people of younger age, male gender, and diverse sociocultural backgrounds.

#### 1.B. Facilitators

Both professionals and patients recognized the key role played by the presence of **1.B.1 Qualified exercise professionals specializing in oncology**. Exercise specialists were said to be in short supply and high demand. They were especially crucial in facilitating patients’ transition out of the in-hospital physical activity program to being active in their communities. Participants also stated that implementing a physical activity program within clinical settings needs **1.B.2 Championing by all concerned parties**. Notably, participants shared that “positive pressure” from the clinical team was key to securing plans for a clinical oncology gym. Indeed, passionate clinicians rallied their colleagues to promote physical activity to their patients; and a clear and shared vision at the higher organizational levels was said to be necessary to bring change to standard delivery of care. Some professionals were adamant that including patients with lived experience was essential to program implementation and especially promotion. Finally, many professionals mentioned the need for **1.B.3 Interprofessional collaboration** among exercise specialists, nurses, doctors, and program managers to promote physical activity to people diagnosed with cancer. Participants stressed that having access to the patients’ data was critical for exercise specialists to tailor care accordingly and communicate with clinicians effectively. Managers were understood to be an essential part of the process namely in securing the necessary resources prospectively.

#### 1.C. Suggestions

Many patients called for clinical settings to **1.C.1 Emphasize exercise professionals’ vital role in oncology**. Various professionals stated that exercise specialists should be integrated into clinical teams. Furthermore, communication between all concerned parties should be improved, they said. To that end, participants suggested experts should **1.C.2 Develop tools and training opportunities.** A list of these suggestions is provided in Table 2. Also, professionals wanted to **1.C.3 Measure physical activity program impact**. They argued that solid data with positive short-, medium- and long-term results would help to boost their inclination to promote physical activity to people diagnosed with cancer as well as to secure funding for the program. Lastly, clinicians and managers indicated that accrued scientific data showing physical activity to be safe in given doses and cancer sites would also boost clinicians’ readiness to promote physical activity to their patients.

### 2. Community factors

These factors incorporate participants’ views concerning the influences operating outside the hospital setting on physical activity promotion for people diagnosed with cancer. They describe how private and philanthropic service offer relates to individual socioeconomic levels and suggestions to overcome challenges to equitable service provision beyond the medical milieu. Table 3 presents illustrative quotes to depict these factors.

**Table 3:** Community level factors: quotes from patients (PAT) and professionals (PRO) describing barriers, facilitators, and improvement suggestions

A. Barriers	B. Facilitators	C. Improvement Suggestions
**2.A.1 Sociodemographic inequities** We’re less well equipped when there’s no return to work planned and when there are financial difficulties because outside the system, everything is cost-based. That’s often the answer we get ‘Yes, but what am I going to do?’ So, we refer, and we have resources, we have external resources that are several partners at the moment with us. Except that even though they are very good partners, and they offer a percentage discount to people from the Virage foundation, it’s still expensive for someone who is off work for a long period of time, often who is going to be off work some more or who has just started again. —PRO1	**2.B.1 Interest from the private sector** I think it’s maybe more and more understood that if our employees, for example, of a company that pays into insurance, are overtaxed and don’t have time to have healthy lifestyles. They’re people who are exposing themselves to getting sick. So, on their insurer’s plan. I think that insurers are increasingly encouraging employees to be physically active. —PRO2	**2.C.1 Develop and nurture intersectoral collaboration** Of course, there’s a challenge, as I said there are people who come from the South shore, the North Shore. So, it’s difficult. I’ve already touched on partnerships: once the patient has had an evaluation, perhaps in their community if we had partnerships with the network, it would be easier sometimes to encourage patients. —PRO4
Look, with the chemo I was doing, the clinic on Monday, because I had a PICC line, to clean the PICC line. And on Tuesday I was doing my chemo. So, it was public transportation each time. And I had to pay out of my own pocket. It was quite… Well, I was on social assistance at the time, so it was difficult. —PAT13	We had a slightly disappointing experience with the [private gym]. We had a manager of a dozen [gyms] who was willing to do it. So, we did a test with the [gym] in Boucherville. They did the cardiac rehabilitation. So, we trained their kinesiologist and then once the training was completed, they could put it on their website ‘Training for cardiac, available here. Collaboration with [University]’. It never lifted. It never got up. Maybe the people there didn’t believe in it as much as the manager did. I don’t know. But I think there’s something there. —PRO17	Look, it could be a business model in the form social organizations that could carry that or not. But I think there should be a business perspective of how we take these kinds of programs and then we’re able to do it. And it can stay in the network, but I mean, if it’s not the best scenario… Could an NGO really make sure to really scale up the program? […] I think the first step is to get all the key players around the table. I don’t know right now, you probably have clinicians, researchers, managers, I guess? So, I think that a facilitator would be to have an interdisciplinary and inter-sectoral team that includes managers, and, I think, sooner rather than later, business expertise. —PRO15
**2.A.2 Service availability and quality concerns** I’ve heard and seen things reported by clients that are a bit abhorrent. I don’t trust [private services] that much. That’s why for me, it’s important to develop ties with professionals and to train professionals working with clients with cancer. It’s very important to me. I’m going to have a hard time recommending otherwise. —PRO7	**2.B.2 Involvement of philanthropic organizations** Because [healthcare center] thought a kinesiology program was a fantastic idea. They wanted one. But getting the funding, the resources, sometimes it can take a long time. But with us, it was done in two weeks. So, we can intervene very, very quickly. We have the flexibility of being as they say a foundation with a vocation and unique, I think, so we are very agile, very flexible, and we do customized programs also for the hospitals. So, what we do is always very simple. We sit down with the co-managers. We tell them what we can do. And we ask them to think about it and say, ‘What do you want us to develop in a phase 1 with you?’ And we do it very quickly—PRO16	**2.C.2 Guide turnkey programs** Well, the facilitator is to equip the teams, in how to implement. So, if tomorrow morning, you go to a [clinic] or [elsewhere] and you want to implement this kind of program, give them a turnkey practice guide. […] Hence the importance of the project holders there who stay involved to support the network. —PRO15
Well, the first thing for me is really the professionalism. That’s what concerns me the most. I might not have participated in a course given by people who I wouldn’t have been convinced have specific training in oncology. […] And the fact that since our immune system is weakened, not being in contact with people with whom this isn’t a major concern. —PAT5	It doesn’t affect me directly, but what I would like to see is that the hospital gives more space to the Virage Foundation. You can see that it’s a vision that they have. What I would have liked is that, they already gave a little, but it would have been even better if I could have seen that they gave even more—PAT17	And there’s a tele-mentoring program. We’ve always done it with front-line health professionals. But I think it could be done [for physical activity in cancer care]. You decide how often, it could be once every two weeks. There’s a team of professionals who give a little capsule of knowledge and after that, it’s an exchange with all the participants who submit clinical situations that they’ve encountered and for which they want to have a consultation, to be able to exchange with other people, to have some information. And it’s tele-mentoring, it’s very, very effective. —PRO18

#### 2.A. Barriers

Two clinicians perceived physical activity to be easily accessible in the community, noting it isn’t difficult to find outlets nearby (e.g., private gyms, paid exercise venues, public parks and swimming pools). Nevertheless, most participants mentioned **2.A.1 Sociodemographic inequities** and the financial challenges people diagnosed with cancer face in adopting or maintaining physical activities. Some clinicians said they promoted physical activity in the community only to cancer patients they knew had the financial means to seek exercise professionals. Exercise specialists and physical activity program managers in our sample noted their clientele overrepresented more educated and wealthier individuals. Most professionals mentioned that physical activity outlets were scarce outside of the city, even more so specialized services for people diagnosed with cancer living in rural settings. Also, professionals and patients shared **2.A.2 Concerns about service availability and quality** in the community. High turnover and labor shortage of exercise specialists working in gyms thwarted efforts to develop partnerships with popular private physical activity outlets in the community. Such ties between health professionals and community physical activity outlets were important to participants. The professionals especially wished to be able to monitor their patients’ progress and ensure that exercise plans were adapted accordingly. Lastly, professionals and patients feared that the quality and cleanliness standards in gyms were insufficient for immunocompromised patients.

#### 2.B. Facilitators

Despite the challenges listed above, professionals noted a beneficial **2.B.2 Interest from the private sector**. Namely, private gym owners showed interest in offering reduced rates for people diagnosed with cancer as well as training their staff to better serve this clientele. Participants noted that a select few insurance companies would reimburse certain of these costs granted the treating physicians provided medical clearance or a prescription to exercise. The **2.B.2 Involvement of philanthropic organizations** was praised in facilitating physical activity promotion for people diagnosed with cancer. These entities were said to be strategically poised to offer tailored services thanks to community financial support. Their close ties with and physical presence in the hospital setting granted them the credibility, knowledge and access needed to offer services that can fill service gaps in physical activity promotion.

#### 2.C. Suggestions

In line with all the above barriers and facilitators to physical activity promotion at the community level, participants called for involved parties to **2.C.1 Develop and nurture intersectoral collaboration**. Most professionals especially indicated that the philanthropic sector’s relative decisional and financial autonomy and the potential for partnerships with the private sector could better be harnessed using tools such as an innovative business model and mentoring other exercise specialist teams remotely across the province. They also called for professionals to **2.C.2 Guide turnkey programs**. That is, for ready to implement, detailed, oncology-adapted physical activity promotion programs to be developed and disseminated to interested community settings.

### 3. Sociocultural factors

Lastly, these factors encapsulate participants’ views concerning the how greater societal forces influence physical activity promotion for people diagnosed with cancer. They include influences such as social norms as well as general and medical culture. Table 4 presents illustrative quotes supporting each of these factors.

**Table 4:** Sociocultural level factors: quotes from patients (PAT) and professionals (PRO) describing barriers, facilitators, and improvement suggestions

A. Barriers	B. Facilitators	C. Improvement Suggestions
**3.A.1 Social norms about cancer survivorship and physical activity** No, I don’t think so, because I think that, as I said, there are many patients who are going to be cocooned by their entourage ‘It’s okay, rest… We’ll take care of everything’ etcetera. So, I think that no: people don’t think of that benefit. —PRO12	**3.B.1 Social support for participation in physical activity during cancer treatment** And to have activities. I think it’s the [hospital center] that had activities to kind of bring together, create a network among the patients. I think that could help. People have a lot of, in oncology, like to develop relations, a network because they’re often isolated. So, if there’s networking physical activities, that could be another option. Even activities other than just on site, to organize activities such as walking or outside but the whole group together. —PRO9	**3.C.1 Raise awareness** We often get asked questions about this, and we like to do little happy hour meetings to inform the clientele and especially things that will attract the population and professionals. Perhaps also sensitize family doctors because there are many now: we can’t follow up on all the patients. So, inform the general practitioners. Sometimes, they also have congresses and meetings, so go and present in these congresses the added value of a training program for patients even when it’s a cancer. So, maybe generalize a little more the information and the scientific data that can demonstrate the added value of continuing [physical activity]. People are always aware but sometimes, as I said, there is still a little taboo for people who have cancer —PRO4
I talked about [physical activity] to others who wanted to lose weight. I would tell them, ‘If you want to lose weight so it’s not all flabby, you have to exercise quite a bit, even take a private trainer—private in that case or in a center that helps you’. The ones that could afford it, yeah, I would talk to them about it. The hindrances in physical activity promotion for cancer too, like I was saying, the women: sometimes, some of them were less interested but the men often were more for it. It depends on the women. —PRO19	But there’s another important factor: it’s the people. The other people we meet who have similar backgrounds and who share things. I find it worthwhile for me to go there rather than in a group of people who don’t necessarily have the same background in terms of follow-up and cancer treatments […] Also, there were two of my friends. We got to know each other after our surgery. We were all three at about the same time and we became friends. And then we did the program together. That, too, made a difference for me, and for them. But I think it’s not so rare. —PAT6	Because there’s the whole side that well, you have to educate the population: it’s important to exercise, it’s the population at large. But when it gets to a point when the population is very sick, something’s gotta give. —PRO10
**3.A.2 Transition back to routine in a demanding world** Because I was back at work and my job [occupation]. I went in, the doctor had told me, ‘You’re going to go about it progressively’. I couldn’t do it progressively. A week later, I was full time. And I was very tired. Also, people are selfish, and I wasn’t able to tell people, ‘I have an activity. I have to leave at such and such a time’. No, it was people—all the time, all the time—even if they knew that: ‘Ah, but can you tell us this? Can you do that?’ So, I started showing up late and it discouraged me. On the other hand, the group had started before I joined. So, people were very, very, very fit. And then me, with the hormone therapy and I hadn’t practiced [physical activity] for a while, I had lost a lot. So, between that and the other… I stopped attending—PAT2	**3.B.2 Medical culture embracing adjunct treatments** It hits me all the time, actually. At my age, I’m from a time when the doctors said ‘Well, take care of yourself: stay at home in bed’ and all that. And now, it’s the opposite revolution that we’re seeing and that I believe has a very beneficial effect as much for recovery as for the return to work or all that. —PRO3	**3.C.2 Standardize universal physical activity cancer care** Well, I think [physical activity] should be built in [to treatment] from the beginning like I said. So, already talking to the patient about it and emphasizing the importance and saying that it’s accessible. I think for it to be integrated into the care program to just say it’s part of the care program. —PRO12
If it had been at the [hospital center] directly, I would have done it. But since I had two places and there was a shuttle between the two… For me, it was really impractical. I have a family life: I had to keep things moving! So, I didn’t give myself the opportunity. —PAT17	We implemented a program that you may know called ERAS. It’s a whole way of thinking that started a few years ago that we integrated here which is a rapid recovery movement. That means that we try to get postoperative patients up immediately, etc. So, it goes against ‘Rest, you’ll see, it will get better’. It’s exactly the opposite. It’s a worldwide movement. —PRO8	And also to make sure that in the oncology center, in all the oncology centers in Quebec, or the surrounding area, that physical activity can be prescribed under the direction of a kinesiologist in a structured program for oncology patients. —PRO13
**3.A.3 Lack of funding for physical activity in cancer care** I was in occupational therapy here through my work insurance. They forced me to do occupational therapy here, but they had no consideration for what I was doing—the kinesiologist: what he had me do. For them, it didn’t matter. I deplore that. It’s the non-recognition of private insurers for these programs. I found that really disappointing. —PAT9	**3.B.3 Population increasingly seeking health information** Because nowadays, patients also ask a lot of questions. So, sometimes we’re very equipped and other times, not so much. So interprofessional collaboration is very important. So, as I just mentioned, having the doctor’s approval, well, if there are specific questions that patients will ask about exercises that I don’t always have the answers to, at that point, it’s reassuring to know that the kinesiologist can inform us about that. —PRO13	**3.C.3 Fund physical activity programs in cancer care** The government must… something has to be done. […] I think it’s as important as having the right chemotherapy protocol. That’s how I see it. And the other thing too is that it’s always a marketable value. I have no control over that. I find that there’s a kind of injustice in the sense that not everyone can have the same services to do [physical activity]. I find that unfair because doing exercise doesn’t require millions of dollars in investment. It costs a lot, but you know, compared to chemo treatment, an operating room, a hospitalization that lasts 10–15 days for a patient who has completely regressed: it’s nothing! It’s nothing: it’s prevention. —PRO10
The main [barrier], the first one: we think of the financial one. It’s the crux of the matter everywhere. We talked to colleagues who start other programs in Quebec City, Sherbrooke … not far. Everywhere, it’s really financial resources that are a problem. —PRO1	People want to hear about physical activity, nutrition, […] So it’s very popular. And the foundation is sensitive to that because they can raise funds with that. —PRO17	[Patients should pay] at least half. You don’t want [patients] to pay zero because if you pay zero, you don’t go to your classes. You know that I mean? And when you pay a lot of money, it encourages you to go. Because you say, ‘It’s coming out of my pocket: I’m gonna go’. […] Kind of like how we save money for our kids because you pay $1,000 and the government gives you $500. So, that’s how people put the money otherwise… Yeah something like that. And parking stubs for sure. For sure they won’t come if there are no parking stubs. — PRO14

#### 3.A. Barriers

Participants named many **3.A.1 Social norms about cancer survivorship and physical activity** that hinder physical activity promotion among people diagnosed with cancer. Namely, people diagnosed with cancer are viewed as fragile, needing passive rest and treatment. In other words, cancer treatment and physical activity can be perceived as antithetical. Moreover, participants noted that physical activity is often considered tedious and secondary to everyday life tasks. These views held particularly true regarding women, whom some clinicians noted were less interested in physical activity. Many women patients shared the challenges of remaining active while also attending to family duties expected of mothers. These social expectations were said to be exponential when in **3.A.2 Transition back to routine in a demanding world**. Participants shared that patients feel pressure from their surroundings to quickly return to their previous self and routine before cancer. These social obligations were said to compete with time spent being physically active. This was especially true about patients returning to work. Finally, most participants perceived a **3.A.3 Lack of funding for physical activity in cancer care**. In the private sector, they said, insurers are slow to fully recognize physical activity participation as a valid health reimbursement. Many professionals felt that prevention was underutilized in healthcare, minimizing the potential physical activity promotion could have on population health. Moreover, there is a lack of government engagement, many participants said, in funding physical activity promotion for people diagnosed with cancer and lack of funding in health in general. For example, one participant witnessed an ongoing debate between the health and the sports and leisure ministries to relinquish funding to another department, if it should ever occur.

#### 3.B. Facilitators

In contrast, participants noted that general **3.B.1 Social support for participation in physical activity during cancer treatment** greatly facilitates physical activity promotion to people diagnosed with cancer. Having a social environment that endorses of physical activity and gaining every day instrumental support helps making it an achievable priority. The same was said of **3.B.2 Medical culture gradually embracing adjunct treatments**. Many participants felt that professionals of all disciplines were progressively more inclined to support complementary forms of treatment and thus to promote physical activity. Lastly, many participants noted that the **3.B.3 Population increasingly seeking health information** increased general awareness of the benefits of physical activity.

#### 3.C. Suggestions

Considering sociocultural barriers and facilitators to physical activity promotion for people diagnosed with cancer, participants suggested **3.C.1 Raise public awareness**. Specifically, they called for raising awareness about physical activity’s benefits to cancer treatment and especially about exercise specialists’ important role in that. Professionals suggested using information technology to equitably target diverse groups of individuals. Second, many participants wished to **3.C.2 Standardize universal physical activity cancer care**. They suggested integrating physical activity into standard cancer care practices province-wide so that all people diagnosed with cancer may have the best and most complete form of treatment. Finally, participants called for greater and shared **3.C.3 Funding physical activity programs in cancer care** for people diagnosed with cancer. Some clinicians stated that individuals should take on the cost of their physical activity during cancer care because the cost to society would be too great otherwise. Some participants suggested that insurance companies be made to cover more costs associated with physical activity participation in cancer care. The greater part of participants felt that it was a governmental responsibility to fund physical activity promotion as a holistic approach to health.

## Discussion

The present study aimed to ascertain patient and professional perspectives regarding physical activity promotion by identifying barriers and facilitators to physical activity promotion and suggestions for its improvement based jointly on patient- and professionals’ experiential and professional knowledge. We identified 24 factors (barriers, facilitators, and improvement suggestions) across the organizational, community, and social levels. We discuss each factor level broadly below.

### Organizational level

A recent scoping review of organizational barriers to promoting physical activity, more specifically integrating structured exercise into cancer care, suggests that organizational barriers account for the larger part of challenges to physical activity promotion [21]. Our results converge with a host of studies showing that clinicians need a better knowledge of evidence-based guidelines on physical activity for cancer population (e.g. ACSM – American College of Sports Medicine Guidelines for Exercise and Cancer, ASCO - American Society for Clinical Oncology Clinical Practice Guidelines), access to clear and simple physical activity promotion tools, a universal referral pathway with standardized workflow tools, as well as champions to advocate for additional resources and to develop collaborative physical activity promotion in interdisciplinary clinical teams [15, 18, 21]. Managers in our sample supported previous work highlighting the importance of patient engagement in program development, implementation, and maintenance [25]. They were adamant about including patients as advocates about the program, especially in the initial phases of development to better coopt decision makers and funders. Our study shows signs of organizational readiness to integrate exercise specialists into interdisciplinary teams, as many clinicians explicitly made this suggestion. Because it could impact potential for program scale-up, it would be interesting to investigate whether this readiness was unique to this setting, the health system in which it exists or even the general population culture surrounding it. Some participants in this study also mentioned the need for physical activity programs designed for specific groups such as young adults, male gender, and diverse sociocultural backgrounds. Physical activity has been less researched in specific groups such as young adults diagnosed with cancer [33]. Tailoring physical activity programs to these groups might fill gaps in current research and practice, and thus achieve more optimal effects on the health and quality of life of these groups. Most importantly, our data shows urgency in showcasing the role that exercise specialists trained in oncology play in the health and survivorship of people diagnosed with cancer. Indeed, as pointed out by study participants, Virage exercise specialists trained in oncology accompany patients from as early as first diagnosis, through treatment and recovery, offering support and working collaboratively to foster a healthier future for patients. Much work needs to be done to highlight their importance and untapped potential in cancer care.

### Community level

This study’s results show how crucial philanthropic entities are to program delivery of physical activity among people diagnosed with cancer. The Virage Foundation’s cost-free physical activity program fostered known levers to participation such as access to no-cost, tailored programs, and available educational materials [15, 19]. It also allowed cancer patients to circumvent certain barriers to participation in physical activity like lack of exercise specialists trained in oncology [18, 19, 21]. Our results additionally highlight opportunities, but also challenges for much-needed community development. Deepenbusch et al.’s review (2021) showed that the quality of the local environment was not a main deterrent of physical activity for people diagnosed with cancer [18]. Rather, in line with our data, lack of service offers specifically tailored for this population was a main barrier to their physical activity participation [18]. Furthermore, in their review, Elshasat et al. (2021) observed that people diagnosed with cancer preferred participating in physical activity at home, in fitness centers, and in hospital centers (in that order) [19]. Fitness centers being important opportunities for structured, supervised physical activity, improving ties with the private industry, and building its community capacity appear to be promising avenues [25].

The organizational managers and leaders in our sample emphasized the potential that well laid-out development plans based on business models could have in improving program sustainability and scale-up. Our data also show that collaboration with franchised fitness centers was hindered namely by high staff turnover despite there being interest from private managers. Moreover, most participants in our study noted that, even at reduced cost, private fitness centers were financially or geographically out of reach for a large number of people diagnosed with cancer. Addressing the concerns of accessibility, especially for rural residents, it is crucial to acknowledge barriers such as distance from physical activity outlets, transportation needs, and individual functional ability. Remotely delivered physical activity interventions have shown promise to mitigate these challenges. Studies have shown the efficacy of interventions using technologies like text messaging, wearable activity monitors, and online coaching [34], videoconferencing [35], as well as remote unsupervised exercise strategies [36] to successfully promote physical activity to people diagnosed with cancer. The integration of these technologies can enhance accessibility, providing tailored support and enabling people diagnosed with cancer in rural areas to engage in physical activity safely and independently, thus bridging the gap in access to community-based programs. To reduce these inequalities in access to physical activity for people diagnosed with cancer, our data points to the potential of applying entrepreneurial strategies to implementation science in guiding program development and maintenance on top of fostering remote video conferencing sessions in line with recent work [9]. Future research on this topic is clearly warranted.

### Social level

Finally, our study sheds light on a crucial aspect of promoting physical activity for people diagnosed with cancer: patients and professionals’ perspectives on its wider social issues. Calls for government financial support for programs or incentives encouraging physical activity promotion for people diagnosed with cancer are widely reported [18, 19, 21]. Participants in the present study expressed ambiguous views concerning who should fund physical activity participation for people diagnosed with cancer. Some studies indicate a clear need for investment in government policies for universal physical activity promotion to people diagnosed with cancer [21, 25]. Given that public support for public policy increases its success rates, it appears relevant that future research should ascertain the general population’s support for such policies. Prioritizing health communication and education as well as promotion could also facilitate policy change implementation [25]. In line with previous work [15, 18], a few health professionals did not consider physical activity promotion to be in the scope of their medical practice, despite the evidence base supporting physical activity as an effective strategy to reduce cancer and treatment side effect and improve physical and mental health. Some health professionals interviewed shared that physical activity was not suitable for people diagnosed with cancer, at certain stages of cancer, or certain types of cancer. This could be due to limited knowledge of physical activity guidelines for cancer patients, perceptions and attitudes towards physical activity and cancer, or lack of tools and referral pathways to promote physical activity [37]. Our study further highlights how social norms and public perceptions concerning physical activity, cancer recovery and even gender roles and work performance shape how we consider physical activity as treatment for cancer despite the growing body of literature on the subject. Social perception studies and social marketing campaigns could be of great benefit to lay the groundwork for physical activity in cancer care to be included as standard cancer care and, thus, for the latest evidence base to be implemented.

Despite our efforts to recruit a diversity of profiles, our patient sample included mostly women with breast cancer, and older middle-aged participants who were active prior to diagnosis, data representativeness is limited. More work is needed to gather data among men, younger adults, and people from diverse ethnocultural groups. Along the same lines, we recruited patients having completed the physical activity program. It is likely that these individuals were the most motivated to be physically active. Further research should investigate structural barriers to physical activity promotion among people diagnosed with cancer who did not participate in such programs. Similarly, we focused on recruiting health professionals with experience in physical activity promotion to increase the opportunity of gaining insight into this practice. However, including those having not engaged in physical activity promotion could have produced more information about barriers to physical activity promotion. Lastly, although our study did include organizational managers, which allowed us to collect critical data, a larger sub-sample of these professionals as well as policymakers and health and fitness industry spokespersons could have allowed us to garner even more insightful perspectives. Future research should aim to fill this gap.

## Conclusion

This study provides a list of 24 factors including barriers, facilitators, and improvement suggestions to consider in a strategy for promoting physical activity in cancer care in Quebec, Canada. It sheds light on the gaps and the bright lights in physical activity promotion for people diagnosed with cancer. Healthcare institutions and policymakers could better implement guidelines and develop a standardized process to direct all cancer patients to appropriate physical activity resources tailored to their needs. Our results suggest that to improve cancer care, it is necessary to showcase exercise specialists as a healthcare resource, to champion for this change within health organizations, to develop partnerships between public and private sectors of the health and fitness industry, and to reassess social standards concerning cancer survivorship and treatment. Scholars have suggested adopting multifaceted strategies that address various barriers to program implementation in clinical settings (e.g., combining social marketing, communication, partnership, and environmental transformations) despite these strategies having shown limited success in increasing physical activity participation in the general population [9]. Our data indicate that more attention should be paid to deconstructing social norms about cancer survivorship and treatment accessibility, physical activity, and gender roles and to constructing an agenda to support people diagnosed with cancer.

## Data Availability

To protect participants’ anonymity, study data are not available. Inquiries concerning study data should be directed to Lise Gauvin, PhD, lise.gauvin.2@umontreal.ca.

## References

[1] Brenner DR, Poirier A, Woods RR, et al. Projected estimates of cancer in Canada in 2022. Can Med Assoc J. 2022;194(17):E601–7. 10.1503/cmaj.212097. 35500919 10.1503/cmaj.212097 PMC9067380

[2] Kerr J, Anderson C, Lippman SM. Physical activity, sedentary behaviour, diet, and cancer: an update and emerging new evidence. Lancet Oncol. 2017;18(8):e457–71. 10.1016/S1470-2045(17)30411-4. 28759385 10.1016/S1470-2045(17)30411-4 PMC10441558

[3] Sung H, Ferlay J, Siegel RL, et al. Global cancer statistics 2020: GLOBOCAN estimates of incidence and mortality worldwide for 36 cancers in 185 countries. CA Cancer J Clin. 2021;71(3):209–49. 10.3322/caac.21660. 33538338 10.3322/caac.21660

[4] Stamatakis E, Ahmadi MN, Friedenreich CM, et al. Vigorous intermittent lifestyle physical activity and Cancer incidence among nonexercising adults: the UK Biobank Accelerometry Study. JAMA Oncol. 2023;9(9):1255–9. 10.1001/jamaoncol.2023.1830. 37498576 10.1001/jamaoncol.2023.1830 PMC10375384

[5] Brown JC, Ligibel JA. Putting exercise into oncology practice: state-of-the-science,iInnovation, and future directions. Cancer J (United States). 2019;25(5):316–9. 10.1097/PPO.0000000000000397. 10.1097/PPO.0000000000000397 PMC6815198 31567458

[6] Berra K, Rippe J, Manson JE. Making physical activity counseling a priority in clinical practice: the time for action is now. J Am Med Assoc. 2015;314(24):2617–8. 10.1001/jama.2015.16244. 10.1001/jama.2015.16244 26662069

[7] Campbell KL, Winters-Stone K, Wiskemann J, et al. Exercise guidelines for cancer survivors: consensus statement from international multidisciplinary roundtable. Med Sci Sports Exerc. 2019;51(11):2375. 10.1249/MSS.0000000000002116. 31626055 10.1249/MSS.0000000000002116 PMC8576825

[8] Das P, Horton R. Physical activity-time to take it seriously and regularly. Lancet. 2016;388(10051):1254–5. 10.1016/S0140-6736(16)31070-4. 27475269 10.1016/S0140-6736(16)31070-4

[9] Purdy GM, Venner CP, Tandon P, McNeely ML. Feasibility of a tailored and virtually supported home exercise program for people with multiple myeloma using a novel eHealth application. Digit Heal. 2022;8:20552076221129064. 10.1177/20552076221129066. 10.1177/20552076221129066 PMC9554139 36249481

[10] Bullard T, Ji M, An R, Trinh L, Mackenzie M, Mullen SP. A systematic review and meta-analysis of adherence to physical activity interventions among three chronic conditions: cancer, cardiovascular disease, and diabetes. BMC Public Health. 2019;19(1):1–11. 10.1186/s12889-019-6877-z. 31126260 10.1186/s12889-019-6877-z PMC6534868

[11] IJsbrandy C, Ottevanger PB, Gerritsen WR, van Harten WH, Hermens RPMG. Determinants of adherence to physical cancer rehabilitation guidelines among cancer patients and cancer centers: a cross-sectional observational study. J cancer Surviv. 2021;15(1):163–77. 10.1007/s11764-020-00921-8. 32986232 10.1007/s11764-020-00921-8 PMC7822788

[12] Nutbeam D, Muscat DM. Health Promotion Glossary 2021. Health Promot Int. 2021;36(6):1811. 10.1093/heapro/daab067. 33979441 10.1093/heapro/daab067

[13] Agency for Healthcare Research and Quality. Five Major Steps to Intervention (The 5 A’s). *Rockville, MD*. Published online 2012. https://www.ahrq.gov/prevention/guidelines/tobacco/5steps.html

[14] Flottorp SA, Oxman AD, Krause J, et al. A checklist for identifying determinants of practice: a systematic review and synthesis of frameworks and taxonomies of factors that prevent or enable improvements in healthcare professional practice. Implement Sci. 2013;8(1):1–11. 10.1186/1748-5908-8-35. 23522377 10.1186/1748-5908-8-35 PMC3617095

[15] Albert FA, Crowe MJ, Malau-Aduli AEO, Malau-Aduli BS. Physical activity promotion: a systematic review of the perceptions of healthcare professionals. Int J Environ Res Public Health. 2020;17(12):4358. 10.3390/ijerph17124358. 32570715 10.3390/ijerph17124358 PMC7345303

[16] Kirkham AA, Van Patten CL, Gelmon KA, et al. Effectiveness of oncologist-referred exercise and healthy eating programming as a part of supportive adjuvant care for early breast cancer. Oncologist. 2018;23(1):105–15. 10.1634/theoncologist.2017-0141. 28982801 10.1634/theoncologist.2017-0141 PMC5759818

[17] Granger CL, Denehy L, Remedios L, et al. Barriers to translation of physical activity into the lung cancer model of care. A qualitative study of clinicians’ perspectives. Ann Am Thorac Soc. 2016;13(12):2215–22. 10.1513/AnnalsATS.201607-540OC. 27689958 10.1513/AnnalsATS.201607-540OC

[18] Depenbusch J, Wiskemann J, Haussmann A, et al. Impact and determinants of structural barriers on physical activity in people with cancer. Int J Behav Med. 2022;29(3):308–20. 10.1007/s12529-021-10014-0. 34550527 10.1007/s12529-021-10014-0 PMC9166881

[19] Elshahat S, Treanor C, Donnelly M. Factors influencing physical activity participation among people living with or beyond cancer: a systematic scoping review. Int J Behav Nutr Phys Act. 2021;18(1):1–20. 10.1186/s12966-021-01116-9. 33823832 10.1186/s12966-021-01116-9 PMC8025326

[20] Huijg JM, Gebhardt WA, Verheijden MW, et al. Factors influencing primary health care professionals’ physical activity promotion behaviors: a systematic review. Int J Behav Med. 2015;22(1):32–50. 10.1007/s12529-014-9398-2. 24788314 10.1007/s12529-014-9398-2

[21] Kennedy MA, Bayes S, Newton RU et al. Implementation barriers to integrating exercise as medicine in oncology: an ecological scoping review. J Cancer Surviv Published Online 2021:1–17. 10.1007/s11764-021-01080-0 10.1007/s11764-021-01080-0 PMC9300485 34510366

[22] Nadler M, Bainbridge D, Tomasone J, Cheifetz O, Juergens RA, Sussman J. Oncology care provider perspectives on exercise promotion in people with cancer: an examination of knowledge, practices, barriers, and facilitators. Support Care Cancer. 2017;25(7):2297–304. 10.1007/s00520-017-3640-9. 28258503 10.1007/s00520-017-3640-9

[23] August M. It’s all about power and you have none: the marginalization of tenant resistance to mixed-income social housing redevelopment in Toronto, Canada. Cities. 2016;57:25–32. 10.1016/j.cities.2015.12.004.

[24] Park J, Lee J, Oh M, et al. The effect of oncologists’ exercise recommendations on the level of exercise and quality of life in survivors of breast and colorectal cancer: a randomized controlled trial. Cancer. 2015;121(16):2740–8. 10.1002/cncr.29400. 25965782 10.1002/cncr.29400 PMC5025035

[25] Schmitz KH, Campbell AM, Stuiver MM, et al. Exercise is medicine in oncology: engaging clinicians to help patients move through cancer. CA Cancer J Clin. 2019;69(6):468–84. 10.3322/caac.21579. 31617590 10.3322/caac.21579 PMC7896280

[26] Kauffeldt KD. It has to be more Than Exercise: exploring optimal physical activity program delivery for breast Cancer survivors across multiple stakeholder groups. Published online 2018. http://hdl.handle.net/1974/24928

[27] Canestraro A, Nakhle A, Stack M, et al. Oncology rehabilitation provision and practice patterns across Canada. Physiother Can. 2013;65(1):94–102. 10.3138/ptc.2011-53. 24381389 10.3138/ptc.2011-53 PMC3563384

[28] Patton MQ. Qualitative Research and methods: integrating theory and practice. SAGE; 2015.

[29] Elo S, Kyngäs H. The qualitative content analysis process. J Adv Nurs. 2008;62(1):107–15. 10.1111/j.1365-2648.2007.04569.x. 18352969 10.1111/j.1365-2648.2007.04569.x

[30] Doré I, Plante A, Bedrossian N, et al. Developing practice guidelines to integrate physical activity promotion as part of routine cancer care: a knowledge-to-action protocol. PLoS ONE. 2022;17(8):e0273145. 10.1371/journal.pone.0273145. 35969619 10.1371/journal.pone.0273145 PMC9377590

[31] Saunders B, Sim J, Kingstone T, et al. Saturation in qualitative research: exploring its conceptualization and operationalization. Qual Quant. 2018;52:1893–907. 10.1007/s11135-017-0574-8. 29937585 10.1007/s11135-017-0574-8 PMC5993836

[32] St-Cyr J, Saint-Onge K, Doré I, Gauvin L. Milestones and turning points in the experience of physical activity throughout cancer care: a qualitative study to inform physical activity promotion. Support Care Cancer. 2023;31(12):682. 10.1007/s00520-023-08093-8. 37943370 10.1007/s00520-023-08093-8 PMC10635913

[33] Brunet J, Wurz A, Shallwani SM. A scoping review of studies exploring physical activity among adolescents and young adults diagnosed with cancer. Psychooncology. 2018;27(8):1875–88. 10.1002/pon.4743. 29719077 10.1002/pon.4743

[34] Gell NM, Grover KW, Savard L, Dittus K. Outcomes of a text message, Fitbit, and coaching intervention on physical activity maintenance among cancer survivors: a randomized control pilot trial. J Cancer Surviv. 2020;14(1):80–8. 10.1007/s11764-019-00831-4. 31776849 10.1007/s11764-019-00831-4

[35] Leach HJ, Crisafio ME, Howell MJ, Nicklawsky A, Marker RJ. A Group-Based, videoconference-delivered physical activity program for Cancer survivors. Transl J Am Coll Sport Med. 2023;8(2). 10.1249/tjx.0000000000000221. 10.1249/tjx.0000000000000221 PMC10653091 37974897

[36] Pelosi AC, Rostirola GC, Pereira JS, et al. Remote and unsupervised Exercise strategies for improving the physical activity of Colorectal Cancer patients: a Meta-analysis. Healthcare. 2023;11(5). 10.3390/healthcare11050723. 10.3390/healthcare11050723 PMC10000866 36900728

[37] Alderman G, Semple S, Cesnik R, Toohey K. Health Care professionals’ knowledge and attitudes toward physical activity in Cancer patients: a systematic review. Semin Oncol Nurs. 2020;36(5):151070. 10.1016/j.soncn.2020.151070. 33010981 10.1016/j.soncn.2020.151070

